# Assessing the Effects of Desertification Control Projects from the Farmers’ Perspective: A Case Study of Yanchi County, Northern China

**DOI:** 10.3390/ijerph17030983

**Published:** 2020-02-05

**Authors:** Xuan Wei, Lihua Zhou, Guojing Yang, Ya Wang, Yong Chen

**Affiliations:** 1 Northwest Institute of Eco-Environment and Resources, Chinese Academy of Sciences, Lanzhou 730000, China; wei_liu_xuan@yeah.net (X.W.); ygj7518@163.com (G.Y.); wy_2018dream@yeah.net (Y.W.); chenyong@lzb.ac.cn (Y.C.); 2 Institutes of Science and Development, Chinese Academy of Sciences, Beijing 100190, China; 3 University of Chinese Academy of Sciences, Beijing 100049, China

**Keywords:** desertification control, project assessment, farmer participation, Grazing Prohibition Project, agro-pastoral ecotone, China

## Abstract

Desertification has inflicted severe damage on the natural environment and social economy for decades, particularly in the arid and semi-arid regions of northern China. In Yanchi County, a series of projects were implemented to combat desertification after 2000. To assess the effects of these Desertification Control Projects from the farmers’ perspective, we divided Yanchi County into two regions (the northern and southern regions) according to their different environmental conditions. We collected data including basic family information, farmers’ perceptions and attitudes, and farmers’ suggestions, in a questionnaire investigation following the Participatory Rural Appraisal approach. Data analysis using the Mann–Whitney U test and Fisher’s exact test revealed that the Desertification Control Projects were generally successful, as the local environment and farmers’ incomes were both improved. Farmers were all satisfied with the effects of the projects, but the farmers in the southern region had a higher acceptance of the projects than those in the northern region. In addition, three problems with the Desertification Control Projects were noted: the farmers had a low degree of participation in the projects, the farmer’s low incomes affected the sustainability of the projects, and the implementation of the complete grazing ban had several adverse effects. We provided suggestions for solving these problems. Our findings have important implications for assessing the effects of environmental conservation projects, especially from a participant’s perspective.

## 1. Introduction

Desertification is a global land degradation problem, severely threatening human survival and development. It is estimated that about 1.9 billion hectares of land and 250 million people are currently affected by desertification worldwide [1]. In response to the threat, many countries have implemented desertification control projects (DCPs), such as the Prairie States Forestry Project in the United States and the Spanish National Action Programme to Combat Desertification in Europe [2,3]. The United Nations Convention to Combat Desertification (UNCCD) came into force in 1996, and proposed a new sustainable development goal for Rio+20—zero net land degradation by 2030 [4].

Many studies have assessed the effects of the DCPs, undertaken by employing diverse theories and methods [5]. Zhang et al. analyzed the normalized difference vegetation index (NDVI) retrieved from satellites for 32 years in northern China, finding that the DCPs contributed to a regional vegetation growth and greening trend [6]. Jafarian et al. used two spatial analysis models and two-dimensional matrix methodology to assess the effects of planting trees in the semi-arid areas in Iran, where forestation was shown to reduce the area classed as “very severe desertification” [7]. In addition, numerous previous studies gauged the effects of the DCPs spanning different time and space scales [8]. Da Silva et al. took a semi-arid zone within northeastern Brazil as the study area, concluding that vegetation gain and the conversion of land use type decreased the rate of soil loss between 1987 and 2010 [9]. Recanatesi et al. studied the changes in soil vulnerability and landscape degradation for 50 years in Italian agro-forest districts. Here, the extensive agricultural system mitigated the desertification risk [10]. Although these studies have identified the effects of the DCPs from different viewpoints, they are not sufficient in revealing the operating mechanism and potential problems of the DCPs. Firstly, most places where the DCPs have been implemented are inhabited by farmers. The measures are also adopted by or upon the farmers. Therefore, unlike natural indices, information from the farmers affords a more human-oriented and practical demonstration of the effects. Secondly, no individual area is uniform and homogenous throughout. Varying outcomes due to intra-regional differences are likely. Potential limitations of the DCPs and concomitant countermeasures may become evident.

China is amongst the countries suffering the most from desertification. Here, desertification has affected the vast arid and semi-arid regions of northern China. Through the processes of wind erosion and salinization, desertification has caused many detrimental effects on the environment and social economy [11]. A series of desertification control projects (DCPs) represent China’s great efforts to combat desertification, such as the Grain for Green Project, the Natural Forest Conservation Project, and the Beijing–Tianjin Wind and Sand Source Control Project [12,13]. At the same time, many lower-level governments have also carried out a number of projects to control desertification on the basis of regional characteristics, which are complementary to the central government’s projects [14]. All the DCPs have achieved desertification control, especially in terms of the increase of vegetation coverage [15]. Scientists have also carried out research assessing the effects of these DCPs [16,17].

Yanchi County is a favorable research field for assessing the effects of the DCPs from the farmers’ perspective. To begin with, Yanchi County is situated in the agro-pastoral ecotone of northern China and suffers from the severe desertification caused by inappropriate human activities. To combat desertification, several considerable DCPs were carried out in Yanchi County after 2000. We note that the Grazing Prohibition Project was initiated throughout the county from 2002, even though Yanchi is famous for its livestock products. Finally, Yanchi County can be divided into the northern region and southern region. The two regions are under the implementation of the same DCPs but have different natural environments, and the farmers also have dissimilar production patterns.

**Figure 1:**
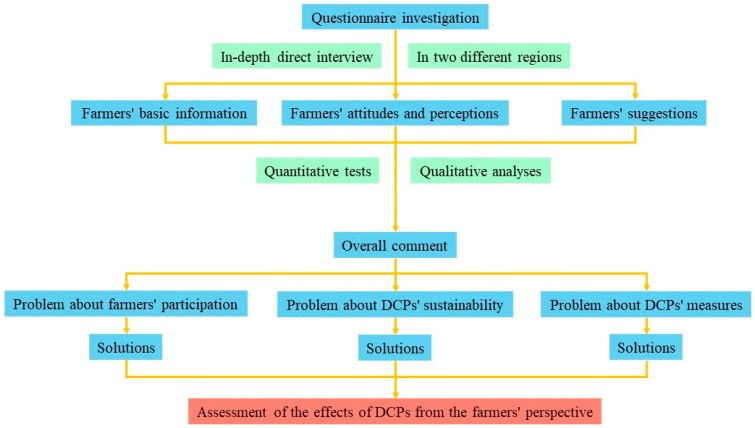
Conceptual framework for assessing the effects of the desertification control projects (DCPs) from the farmers’ perspective.

The purpose of this paper was to adopt an optimal approach to assessing the effects of the DCPs implemented in Yanchi County in Northern China after 2000. The conceptual framework of the paper is shown in Figure 1. In our assessment of the DCPs, we attached great importance to the information obtained directly from the farmers, such as their attitudes and perceptions. Farmers are one of the key stakeholders in the implementation of the DCPs. The views from the farmers allowed us to explain the effects of the DCPs from a social perspective. We also considered intra-regional differences in the assessment process (particularly the north–south regional differences). We paid attention to both the commonalities and differences between the two regions and the farmers, which led to a comprehensive evaluation of the DCPs. The results of this study will help to assess the effects of the DCPs and other similar environmental conservation projects from the farmers’ perspective.

## 2. Materials and Methods

### 2.1. Study Area

Yanchi County (106°30′–107°41′ E, 37°04′–38°10′ N) lies in the eastern part of Ningxia Hui Autonomous Region and covers a total area of 6774 km^2^, extending nearly 110 km north–south and 66 km east–west. The Mu Us Sandy Land, which adjoins the county to the north, is one of the four major sand areas in China. Yanchi County has a typical temperate continental semi-arid climate, meaning that it has a short summer and long winter, a large temperature difference, and low rainfall but high evaporation [18]. The annual average temperature is 8.7 °C, and the annual temperature range is 28 °C. Annual precipitation is 250–350 mm, mainly concentrated in July and August and decreasing from south to north. In addition, strong winds and drought often occur simultaneously during winter and spring. Yanchi County is known as the home region of China’s Tan sheep (a kind of sheep famous for its delicious mutton and high-quality sheepskin), and is also the main producing area of China’s licorice (a traditional profitable Chinese herbal medicine). Grazing is the main agricultural activity in Yanchi County—in 2017, the income from grazing and livestock production accounted for 56.1% of the total agricultural income [19]. Yanchi County contains eight towns, and in 2017 the total population of the county was about 172,000, of which 84.4% were associated with agriculture [19].

**Figure 2:**
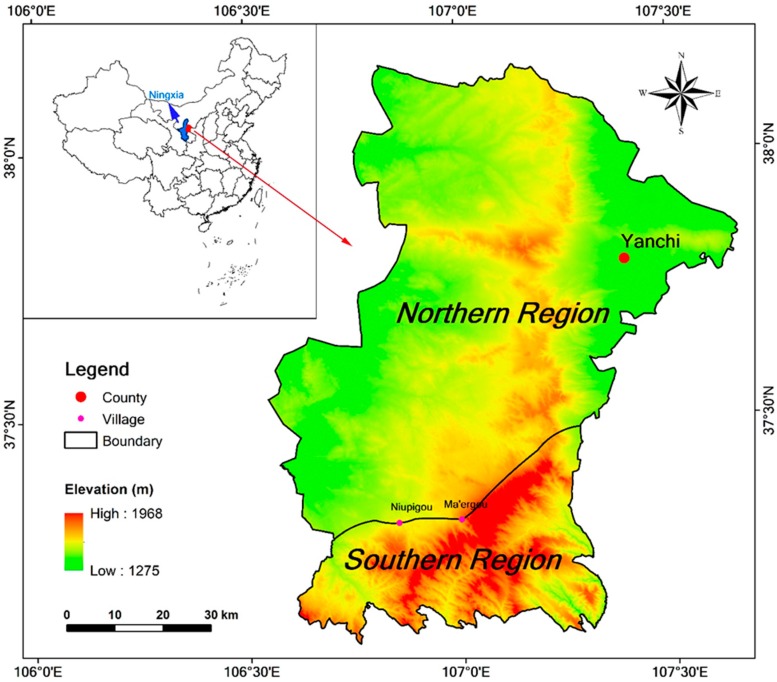
Location of Yanchi County. Ma’ergou and Niupigou are two villages in the boundary between the northern and southern region.

Yanchi County is in the agro-pastoral ecotone of northern China, which is the transitional zone from cropping in the south to grazing in the north [20]. Historically, Yanchi County has for a long time been spatially interlaced with cropland and pasture, with temporally overlapping sedentary agriculture and nomadic pasturing. According to this transition, Yanchi County can be divided into two parts: the northern region and southern region (Figure 2), covering about 78.7% and 21.3% of the total county area, respectively. Although the boundary between the two regions runs roughly along the mountain ridges, there are further differences between them. Firstly, topographically, the northern region comprises hilly Ordos Mesa with gentle slopes, and altitudes mostly under 1600 m. The southern region comprises hilly Loess Plateau and altitudes mostly above 1600 m. Secondly, the vegetation types in the northern region are mostly desert steppe and sand vegetation, and the vegetation type in the southern region is mainly steppe vegetation. Thirdly, the soil types are mainly aeolian sandy soil and sierozem in the north, and dark loessial soil in the south. Fourthly, there is more grassland in the northern region, and thus farmers there raise sheep as the main agricultural activity. The southern farmers do not graze many sheep, and instead have more arable land than the farmers in the north. Lastly, owing to the environmental conditions described above, the northern region is more susceptible to wind and sand hazards than the southern region.

In the past decades, Yanchi County has experienced severe desertification and land degradation mainly caused by over-grazing and excessive digging for licorice. To combat the desertification, three primary DCPs have been implemented. The Grain for Green Project was initiated in 1999, converting the sloping or desertified cropland into forest and grass. The Natural Forest Conservation Project began in 2000, forbidding cutting the natural forests for commercial interests. The Grazing Prohibition Project began in 2002, inhibiting all farmers from grazing sheep in the grassland throughout the whole county; as a result, sheep could only be raised by stall-feeding. All three DCPs are mandatory top-down projects. According to the land area and time period involved affected by the DCPs, rural households were given an appropriate subsidy by the government as compensation. Among these DCPs, the Grazing Prohibition Project was the most significant and had the greatest participation, as it is the only project based on the local conditions of Yanchi County.

### 2.2. Data and Analysis

We conducted a questionnaire survey in October of 2017 to assess the effects of the DCPs from the farmers’ perspective. In this investigation, the Participatory Rural Appraisal approach was used [21]. The most prominent feature of the Participatory Rural Appraisal approach is that the whole process of investigation emphasizes the participation of farmers, and thus the results are more operationally relevant to the farmers. The investigation was conducted by a semi-structured interview based on questionnaires. In the in-depth face-to-face interviews, the questionnaire was designed in advance, but the order of asking the questions and the way of explaining them to the participants were formed during the communication process. The content of the conversation and the answers to the questions supported each other, increasing the credibility of the investigation results. Before the formal investigation, a pilot survey was undertaken in 15 random households, and the questionnaires were improved according to these initial responses. The formal survey covered all eight towns of Yanchi County, and the households were selected by stratified random sampling according to the population of each town. There was no local authority involvement or assistance before and during the interviewing process. Additionally, the farmers were informed that the questionnaires were anonymous and that all the information obtained would only be used for academic purposes. Hence, the answers showed the true, independent view of the farmers. All the participants provided their verbal consent to our investigation.

The designed questionnaire included both multiple-choice and open-ended questions. The multiple-choice questions sought to investigate and characterize the interviewees’ attitudes and perceptions. The open-ended questions aimed to extract the specific reasons for the farmers’ behavior. The questionnaire comprised five sections, based on the five categories of information needed, and was composed of basic family information such as household income, farmers’ perceptions of environmental changes after the DCPs, farmers’ attitudes towards the DCPs, farmers’ environmental awareness, and farmers’ suggestions about the DCPs.

Firstly, the average values or percentages of the responses were calculated to show the general characteristics of the data. Then, the data were analyzed by the Mann-Whitney U test and the Fisher’s exact test. Both test methods were used for detecting whether there was a significant difference between the northern and southern regions. For ordinal categorical data such as income and other basic information, the Mann-Whitney U test [22] was used. For the nominal categorical data such as the answers “yes” and “no”, the Fisher’s exact test [23] was used. The tests were processed by the SPSS 24.0 statistics software (IBM Corp., Armonk, NY, USA). Moreover, we qualitatively analyzed the farmers’ responses to the open-ended questions.

After excluding 8 invalid questionnaires, a total of 212 (response rate of 96.4%) valid questionnaires were collected in the two regions: 165 and 47 from the northern and southern regions, respectively. Among all the respondents, 71.7% were male, and 55.2% of them were in the age range of 40–65 years (middle age); therefore, most of the interviewees were household heads or were well-informed of the household’s affairs, such that the information provided was comprehensive and accurate.

## 3. Results

### 3.1. Basic Family Information

Table 1 summarizes the basic family information about the interviewees. There were about three persons in each household in both regions. With regard to the land, each household in the southern region had an average of 3.44 hectares of cropland, which was more than the average of 2.66 hectares in the northern region. Similarly, the southern region had more land participating in the DCPs than that in the northern region (1.42 hectares per household versus 0.52 hectares per household). The northern region had 39.3 sheep per household, more than the 8.4 sheep per household in the southern region (Table 1). The farmers in the northern region were significantly wealthier than farmers in the southern region, according to the gross income data. It is noteworthy that the per capita net income of the farmers of both regions was lower than the national average [19]. The mean incomes ranked in descending order, for the northern region were non-agricultural income, livestock income, crop income, and subsidy; those in the southern region were non-agricultural income, crop income, subsidy, and livestock income (Table 1). Non-agricultural income accounted for the highest proportion of gross income in both regions, contributing 55.3% and 70.2% in the northern and southern regions, respectively. Most non-agricultural workers did not have a consistent and stable income, and the elderly and women in the villages were rarely able to work. Therefore, it cannot be considered that the non-agricultural income was more important than the crop income or the livestock income. The livestock income was greater than the crop income in the north, but vice versa in the south. In terms of subsidies, the difference between the northern and southern regions was significant. Subsidies as a proportion of gross income were not equal in the two regions: 5.39% in the northern region and 9.90% in the southern region (Table 1).

### 3.2. Farmers’ Attitudes and Perceptions

In both regions, the vast majority of the farmers made a positive evaluation of the environmental changes after implementation of the DCPs. About 97.6% of the farmers in the north and 97.9% of the farmers in the south (figures are quoted in the same order hereon in) believed that the desertification had reversed, and 97.0% and 89.4% of the farmers considered that the vegetation coverage was higher than that before the DCPs (Table 2). Fisher’s exact test showed that there was no significant difference between the two regions in the attitudes towards the changes of the species richness; 48.5% and 63.8% of the farmers deemed that the species richness was higher than before, but 45.5% and 34.0% of the interviewees held the “no change” opinion (Table 2).

Fisher’s exact test indicated that there was no difference in farmers’ understanding of the DCPs between the two regions: less than half of the respondents (50.9% and 38.3%, respectively) were familiar with the DCPs (Table 3). In spite of the significant difference between the two regions according to the Fisher’s exact test, most farmers (81.8% and 97.9%) accepted the DCPs voluntarily, and they were also mostly satisfied (90.9% and 97.9%) with the consequences of the DCPs. Farmers in both regions (58.8% and 74.5%) had a certain understanding of the subsidy standards of DCPs, and significantly more farmers (68.5% and 87.2%) living in the southern region considered the subsidy standards fairly reasonable (Table 3). In both regions, about half of the interviewees (40.0% and 53.2%) argued that the DCPs facilitated an increase in household income, and almost the same proportions (45.5% and 42.6%) reported that income remained the same (Table 3).

With no difference according to the Fisher’s exact test, the majority of the farmers in both regions (95.8% and 97.9%) expressed that their environmental awareness had improved after the DCPs were implemented (Table 4). Most of the respondents (64.2% and 66.0%; no significant difference) argued that environmental protection and economic development were both important (Table 4). The Fisher’s exact test indicated that most farmers (61.8% and 63.8%) in both regions were willing to proceed with the measures controlling desertification if the government’s subsidy ended. However, if investing their own money in those measures, the proportion of unwillingness was larger than that of willingness (60.0% and 53.2% of the farmers were unwilling; Table 4). According to the open-ended question about the reasons for unwillingness, 67.4% of the farmers thought that they could not afford to pay such a large sum of money, and 30.5% believed that it was the government’s responsibility to bear the expense of desertification control.

### 3.3. Farmers’ Suggestions

**Figure 3:**
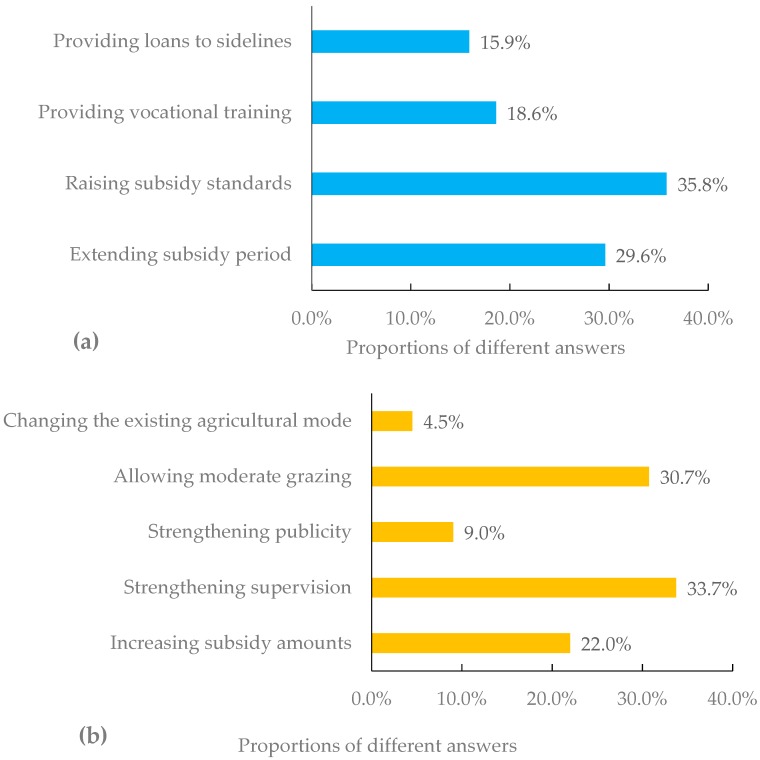
Farmers’ suggestions about the DCPs: (**a**) suggestions for adjusting subsidy policy; (**b**) suggestions for regulating illegal grazing.

The farmers’ suggestions about how the subsidy policy should be adjusted (Figure 3a) were as follows, in descending order: raising subsidy standards (35.8%), extending subsidy period (29.6%), providing vocational training (18.6%), and providing loans to sidelines (15.9%). Therefore, what the farmers wanted most was to increase the total amount of the subsidy they could get. The total amount of the subsidy could be increased by raising the existing subsidy standards or by extending the time period of the subsidy. Regarding how to regulate illegal grazing activities, the farmers’ suggestions (Figure 3b) were, in descending order: strengthening supervision (33.7%), allowing moderate grazing (30.7%), increasing subsidy amounts (22.0%), strengthening publicity (9.04%), and changing the existing agricultural mode (4.52%). It should be noted that a considerable proportion of the farmers proposed the notion of allowing moderate grazing, which was against current regulations. Additionally, many farmers individually proposed diverse suggestions for improving the DCPs, such as the implementation of rotational grazing. Although these suggestions were not included in any multiple-choice questions, they were properly documented.

## 4. Discussion

### 4.1. Overall Comment

Generally speaking, the DCPs carried out in Yanchi County have been successful so far. After the implementation of the DCPs, the local environment was improved (Table 2), and the farmers’ income was increased (Table 3). These two factors are the prerequisites leading to the success of the DCPs, and likewise any other environmental conservation projects. Almost all of the world’s environmental conservation projects are funded by governments or donors rather than the local farmers. The projects care mostly about the improvement of the environment instead of the farmers’ welfare. Nevertheless, the farmers are usually the direct practitioners of the projects and reside in the project areas. The measures of most projects, such as logging bans or grazing bans, will increase the opportunity cost of farmers. Hence, appropriate capital compensation should be given to the farmers to maintain a reasonable standard of living [24]. Accordingly, for any environmental conservation projects, only if both the environment and economy are well improved can the projects be considered as well implemented and as engendering expected influences [25,26]. Many environmental conservation projects have been carried out in various countries around the world, but have failed to fulfill these criteria. For example, the Social Forestry Program (SFP) implemented in the south-west coastal region of Bangladesh did not largely increase local forest cover or improve the target population’s livelihoods, thereby deviating from the original objectives [27]. The Tropical Forest Conservation Project in Madagascar had a similar problem [28].

From the environmental aspect, before 2000, and due to adverse climatic conditions and improper human activities (mainly over-grazing and digging for licorice), the grassland in Yanchi County was severely degraded and the desertification caused serious harm to cropland. After the initiation of the DCPs, grazing and digging for licorice were prohibited in the county, and many shelterbelt forests were planted. It was widely recognized by the farmers that local vegetation coverage obviously increased and that desertification was considerably reversed (Table 2). From the economic aspect, before the implementation of the DCPs, pasturing (in the whole county) and crop-planting (more in the southern region) were the main agricultural activities in Yanchi County, and the farmers had reasonable incomes; however, the number of sheep and the area of cultivated farmland both decreased because of the measures of the DCPs. The government provided the farmers enrolled in the DCPs with a subsidy for their loss of income. According to the questionnaires, the Grain for Green subsidies were 428.9 dollars per hectare per year in the first 8 years, and 193.0 dollars per hectare per year in the following 5 years. For the Grazing Prohibition Project, if the grassland was fenced and met the pre-set criteria, the government paid the farmers 12.9 dollars per hectare per year as compensation. In addition, the government introduced preferential policies to encourage migrant work as a supporting measure for the DCPs. The farmers earned a better non-agricultural income, which contributed the largest proportion in the gross income (Table 1). Finally, the total income of the farmers increased, rather than decreased. In summary, a poor environment usually comes with a poor economy, forming a vicious circle. However, the DCPs in Yanchi County addressed both problems simultaneously; therefore, the living environment improved and the economic income increased, forming a virtuous circle.

### 4.2. Similar Attitudes but A Higher Acceptance in the Southern Region

According to the previous analysis, most farmers in both the northern and southern regions of Yanchi County were satisfied with the effects of the DCPs. However, the Fisher’s exact test indicated that the farmers in the southern region had a significantly higher acceptance of the DCPs (Table 3), perhaps due to two reasons regarding the environment and economy. These two reasons are closely related to the different natural conditions and production activities in the two regions. (1) From the aspect of the environment, the northern region is adjacent to the Mu Us Sandy Land and has been threatened by desertification in past decades. The grassland was seriously degraded in the northern region. Most land in the southern region was non-desertified land. After the implementation of the DCPs, the vegetation coverage in the northern region increased substantially and the desertification was distinctly reversed. In contrast, the southern region did not need nor was able to change substantially, because it already had an adequate degree of environmental greening [29]. Therefore, in terms of vegetation coverage, the proportion of “much better” in the north (75.8%) was significantly larger than that in the south (59.6%). There was an appreciable proportion of “no change” (10.6%) in the southern region (Table 2). Overall, the southern region maintained good vegetation coverage during that whole time. Although the DCPs improved the environment in the north, there was still a vast tract of desertified land that needed to be ameliorated. Thus, farmers in the north would have had a lower acceptance of the DCPs than those in the south. (2) From the economic aspect, the Fisher’s exact test demonstrated that there were no significant differences between farmers’ crop income and non-agricultural income between the two regions, whereas there were significant differences between the livestock income and subsidy. The proportion of earnings from livestock was much greater than that from the subsidy (Table 1). Because the northern region had much greater livestock production, it could be inferred that the measures such as grazing prohibition could have led to a sharp drop in the income of the northern region. The farmers in the southern region did not experience a big loss in the livestock income, and also had a significantly more satisfactory subsidy (Table 3). It is clear that the farmers in the southern region were more supportive than the farmers in the northern region.

No matter where the environmental conservation projects are carried out, the implementation area such as a city or a state will not be a uniform and homogenous entity. Natural and socio-economic differences will exist [30]. The projects need to improve the environment and enhance people’s livelihood, so the measures of the projects will inevitably interact with local conditions. Due to intra-regional differences, the interactions will appear dissimilar; such differences should be taken into consideration when assessing the impacts of the environmental conservation projects. This is consistent with the findings of Shao et al. regarding the Grain for Green Project [31]. In their research, the studied river basin was divided into three regions according to different ecological vulnerability levels. The three regions then were investigated separately. In the present study of Yanchi County, it is known that, for environmental and economic reasons, the acceptance of the DCPs in the northern region was not high enough. Therefore, improvements should be made to the DCPs in the future. Under the premise of overall planning, specific measures and policies should be appropriately weighted towards the northern region—for example, by adjusting the subsidy of grassland or livestock.

### 4.3. The Problem Regarding the Farmers’ Participation and Corresponding Solutions

It was concluded that the DCPs had achieved improvements in the environment and economy, and also gained farmers’ positive attitudes. Nonetheless, several problems were found through the analysis of the questionnaires. The first problem was that the farmers were not familiar with the DCPs. Fewer than half the farmers had a good knowledge of the DCPs (50.9% and 38.3% in the northern and southern regions, respectively). Similarly, it is noteworthy that 41.2% and 25.5% of the farmers did not understand the subsidy standards (Table 3). This revealed that the farmers did not deeply participate in the DCPs, and merely acted as the passive recipients of the projects. Two reasons are able to explain this problem. The first reason concerns the projects themselves. Projects such as the DCPs are mostly nation-centered projects formulated by a top-down approach, of which the government is the primary driver [32]. The central government sets the objectives and the implementation framework of the projects. Moreover, the vast majority of the funds are provided by the government. Therefore, the farmers are treated as one of the stakeholders on the surface, but in fact they have little involvement in the decision-making of the projects [33]. The DCPs implemented in Yanchi County are ongoing examples of this issue. The second reason is the insufficient institutional capacity. Despite not farmers participating in the formulation process of the projects, the farmers were able to be compensated in the later implementation process. However, the department administering the DCPs in Yanchi County was not able to provide good communication channels. The intentions of the government did not adequately reach the farmers through publicity, and the farmers were unable to promptly and conveniently report the problems they found. Eventually, the farmers only blindly “believed in the country” and mechanically obeyed the state’s commands, leading to a low degree of real participation. For the same reasons, the environmental conservation projects in Minqin County also lacked enough public participation [34].

The farmers’ participation plays a vital role in the implementation of the environmental conservation projects and the achievement of corresponding goals. If the communication between the upper and lower administrative levels is smooth, farmers will have the sense of ownership. They will participate in projects proactively and take the projects as their own business rather than the country’s. With a higher participation of the farmers, the projects could be modulated and improved over time. This opinion is in accordance with the findings of research in Vietnam [35], where Van Cuong et al. argued that the locally poorly-developed communication channels caused limited public awareness and understanding of the biosphere reserve approaches. This research also noted that the improved public participation was important to fulfill the aims of the biosphere reserves. The present research in Yanchi County shows that farmers’ participation in the DCPs could be increased by strengthening the publicity work of the local government. Initially, convenient communication conditions for farmers need to be provided, such as useful transportation facilities and online platforms. Additionally, publicity for the DCPs could be available in various forms, such as brochures, meetings, and education [36]. These forms of publicity could make the farmers more knowledgeable about the DCPs and have a sense of actual participation.

### 4.4. The Problem Regarding Sustainability and Corresponding Solutions

The results showed that the environmental awareness of the most farmers (95.8% and 97.9%) had remarkably improved after the implementation of the DCPs. Even if the projects’ subsidies stopped, most of the farmers (61.8% and 63.8%) chose to continue with the measures to combat desertification. Nevertheless, when asked if they were willing to invest their own money in the desertification control, the unwilling people were the majority in both regions (Table 4). This seemingly unreasonable phenomenon is associated with conflicts of interests. The first contradiction is between the short-term interests and the long-term interests. The DCPs were aimed at the ultimate improvement in Yanchi County’s natural environment. To gain the long-term interests, the local government carried out the corresponding measures. However, the realization of the interests would take a long time period, maybe decades or longer. The farmers that participated in the DCPs would have agreed to improve the environment, but this time period was too long for them. On the contrary, they paid more attention to the short-term interests, namely, the steady rise in the standard of living in the foreseeable future [37]. The second contradiction is between the environmental interests and the economic interests. Before 2000, the natural environment in Yanchi County was extremely poor. This had detrimental effects on the living standards and productivity of the farmers. After the implementation of the DCPs, the various benefits brought by the better environment received the attention of the farmers, as demonstrated by their improved environmental awareness (Table 4). However, once the environmental conditions had reached the basic standards, the marginal returns from the environmental improvement were diminishing [38]. At this time, it was the economic interests that were more valued by the farmers. In their opinion, the amount of wealth determined the standard of their family life. After analyzing the two contradictions, the conditions in Yanchi County should be considered. The environment did not deteriorate to an intolerable extent, and the farmers were not wealthy. Therefore, letting the farmers invest their own money to control the desertification is equivalent to exchanging their short-term economic interests for long-term environmental interests. It is clear the farmers would not agree with this exchange.

The two contradictions run through the whole process of the implementation of the DCPs. If the contradictions are not properly resolved, they will inevitably affect the long-term sustainability of the DCPs. From the above analysis, it is obvious that both contradictions arise because of the low income of the farmers. Research by Feng et al. reached similar conclusions [39]; they argued that economic incentives were necessary for farmers to replace unsustainable practices with more sustainable practices. For the improvement of the DCPs in Yanchi County, the following two approaches could be adopted to raise the farmers’ income. First would be devising a subsidy policy that is more conducive to the farmers. Gao et al. proved that the project’s subsidy had a positive impact on increasing farmers’ income [40]. The measures of the DCPs included the reduction of cropland and grazing, causing the loss of agricultural and livestock income. Thus, the subsidy policy could be adopted on the basis of local average income levels. Second would be increasing the income of the existing production activities. For the agricultural income, raising the price of agricultural products and livestock products is one helpful step. For the non-agricultural income, the local government could provide preferential employment regulations, employment opportunities, and vocational skills training.

### 4.5. The Problem with the DCPs’ Measures and Corresponding Solutions

The DCPs were implemented for nearly 20 years in Yanchi County. During this period, there was growing discussion about the measures of the DCPs, especially the Grazing Prohibition Project. Many local farmers proposed opposing views on the present county-wide complete ban on grazing, which were reflected in the questionnaires. The farmers believed that the complete grazing ban had three adverse effects. First, complete grazing prohibition caused the degradation of grassland ecosystems, including the soil, vegetation, and animals. For example, most farmers (97.0% and 89.4%) reported that the grassland vegetation coverage increased after the DCPs, but not a great proportion of the farmers (48.5% and 63.8%) thought that the species richness was higher than before (Table 2). The present finding is consistent with the findings of Pulungan et al., who claimed that an intermediate grazing intensity instead of no grazing often resulted in the highest species diversity [41]. Moreover, the complete grazing ban forced sheep to be only bred in sheep pens. However, forage was difficult to obtain especially in winter. Many farmers also reported that the meat of the sheep raised in the pens was not as good as that of the sheep stocked on the grassland. This is supported by the study of Popova et al. which revealed that pasture rearing was beneficial to meat dietary quality [42]. Furthermore, the strict ban on grazing promoted illegal grazing to some extent. Because the stall-feeding was not able to meet all the needs, some farmers grazed sheep on grassland at night to evade the monitoring personnel. The illegal grazing was a direct violation of the DCPs’ objectives. In short, after nearly 20 years of implementation, the farmers found that the complete and comprehensive ban on grazing was not the best solution.

With regard to the adjustment of the current grazing ban in Yanchi County, farmers proposed some ideas based on local conditions and their own experience. Firstly, restoring the old way of unrestricted grazing is definitely not feasible. There was illegal grazing even under the grazing ban measures. If there were no restrictions on grazing, the grassland would probably be degraded to the previous poor situation. Moreover, rotational grazing and seasonal grazing are not appropriate. Unlike some areas with fewer people and more grassland, such as Inner Mongolia in China [43], Yanchi County has a small area of grassland and large population. The local farmers face high economic pressure, which is not conducive to carrying out rotational grazing or seasonal grazing. Lastly, many farmers argued that the grazing ban should be adjusted to allow moderate grazing. The following two factors are necessary for moderate grazing. Initially, the consolidation of grassland use rights is a prerequisite. If the use rights of grassland were consolidated and each family had their own pasture, the farmers would regard the pasture as a long-term source of income. They would possibly only stock a proper number of sheep, which would be fewer than previously. Additionally, a sufficient subsidy provided by the government is the safeguard. Even if moderate grazing is allowed, the farmers’ livestock income is still much lower than that before the DCPs. The government’s financial support is indispensable. Therefore, the top two suggestions from the farmers about the adjustment to the subsidy policy were raising subsidy standards (35.8%) and extending the subsidy period (29.6%).

## 5. Conclusions

The implementation of the Desertification Control Projects is vital for the arid and semi-arid regions of northern China. By analyzing questionnaire data from Yanchi County, this study assessed the effects of local DCPs carried out after 2000 from the farmers’ perspective. We found that the DCPs had effectively improved the local environment and increased the farmers’ income. The farmers from both regions of Yanchi County had positive attitudes towards the DCPs, but farmers in the southern region had a higher degree of acceptance. Three problems concerning the farmers’ participation, the projects’ sustainability, and the DCPs’ measures were identified, and suggestions for solving these problems were discussed.

This paper adopted a novel approach to assessing the effects of the DCPs from the participants’ perspective. The findings of this study have some important implications for other regions with similar environmental conservation projects, even in other continents or under different socioeconomic conditions. Firstly, all the results are based on the direct interviews with the farmers, which allows us to treat the government projects in a humanistic way. In this study, the farmers’ feelings and opinions are the center of consideration, rather than relying on official comments or corporate profits. Secondly, intra-regional differences exist in all project areas. Assessing the effects of projects on the basis of the intra-regional differences does not complicate the assessment, but instead gives the study a stronger scientific basis and improves its reliability. Finally, the problems found in the DCPs of Yanchi County are a valuable reference. Similar environmental conservation projects in other areas may have similar problems to those of the DCPs, such as the low participation rate. In summary, this paper analyzed the measures and achievements made by China to combat desertification, contributing to the UNCCD and its goal of zero net land degradation. However, compared with the total population of Yanchi County, the number of respondents interviewed in this study is rather small. Some results are tentative in their generalizations and understanding of the farmers’ attitudes. Further research needs to consider the sample size and sample representativeness, which could allow firmer results and suggestions.

## Figures and Tables

**Table 1:** Basic family information of farmers.

Items	Northern Region	Southern Region		
	Mean ± SE	Median (Range)	Mean ± SE	Median (Range)
Persons per household	3.6 (0.1)	3.0 (7.0)	3.1 (0.2)	3.0 (6.0)
Area of cropland (ha per household)	2.66 (0.13)	2.33 (6.20)	3.44 (0.28) **	2.97 (5.20)
Area of land participated in the DCPs (ha per household)	0.52 (0.05)	0.35 (1.20)	1.42 (0.17) **	1.20 (2.73)
Number of sheep (per household)	39.3 (5.2) **	0 (100.0)	8.4 (3.2)	0 (25.0)
Crop income (dollars)	1030.1 (143.0)	0 (2898.6)	570.5 (97.2)	333.3 (1449.3)
Livestock income (dollars)	1320.1 (215.0) *	0 (3478.3)	242.1 (80.0)	0 (579.7)
Non-agricultural income (dollars)	3306.4 (375.2)	660.9 (9941.3)	2872.3 (767.1)	542.2 (6521.7)
Subsidy from the DCPs (dollars)	322.2 (29.9)	218.8 (847.8)	404.8 (46.5) *	347.7 (814.5)
Gross income (dollars)	5978.7 (466.9) *	4521.7 (14,492.8)	4089.7 (788.8)	2394.9 (7296.5)

Notes: All income means net income in dollars per household; asterisks (*) indicate significant difference between regions (*p* < 0.05); double asterisks (**) indicate extremely significant difference between regions (*p* < 0.01); SE means standard error of mean.

**Table 2:** Farmers’ perceptions of environmental changes after the DCPs.

Questions	Answers	Percentage of Responses by Region	
		Northern Region (%)	Southern Region (%)
1. Did the desertification reverse?	1. Reversed a lot	90.9	91.5
	2. Reversed	6.7	6.4
	3. No change	2.4	2.1
2. What is the change of the vegetation coverage of grassland?	1. Much better	75.8 *	59.6
	2. Better	21.2	29.8
	3. No change	3.0	10.6
3. What is the change of the species richness of grassland?	1. Higher	48.5	63.8
	2. Lower	6.1	2.1
	3. No change	45.5	34.0

Note: asterisks (*) indicate significant difference between regions (*p* < 0.05).

**Table 3:** Farmers’ attitudes towards the DCPs.

Questions	Answers	Percentage of Responses by Region	
		Northern Region (%)	Southern Region (%)
1. Are you familiar with the DCPs implemented by the government?	Yes	50.9	38.3
2. Do you accept the DCPs?	Yes	81.8	97.9 *
3. Are you satisfied with the consequences of the DCPs?	Yes	90.9	97.9
4. Are you familiar with the subsidy standards of the DCPs?	Yes	58.8	74.5
5. Do you think the subsidy standards of the DCPs are reasonable?	Yes	68.5	87.2 *
6. What is the influence of the DCPs on your income?	1. Increase	40.0	53.2
	2. Decrease	14.5	4.3
	3. No influence	45.5	42.6

Note: asterisks (*) indicate significant difference between regions (*p* < 0.05).

**Table 4:** Farmers’ environmental awareness after the DCPs.

Questions	Answers	Percentage of Responses by Region	
		Northern Region (%)	Southern Region (%)
1. Will you proceed with the measures controlling desertification if the government’s subsidy ends?	Yes	61.8	63.8
2. Will you invest your own money in the measures of controlling desertification?	Yes	40	46.8
3. What is the change of your environmental awareness?	1. Improved a lot	60.0	70.2
	2. Improved	35.8	27.7
	3. No change	4.2	2.1
4. Which is more important for you (environmental protection or economic development)?	1. Environmental protection	21.2	29.8
	2. Economic development	14.5	4.3
	3. Both important	64.2	66.0
