# Erratum: Xuan Wei, et al. Assessing the Effects of Desertification Control Projects from the Farmers’ Perspective: A Case Study of Yanchi County, Northern China. *Int. J. Environ. Res. Public Health* 2020, *17*, 983

**DOI:** 10.3390/ijerph17228415

**Published:** 2020-11-13

**Authors:** Xuan Wei, Lihua Zhou, Guojing Yang, Ya Wang, Yong Chen

**Affiliations:** 1 Northwest Institute of Eco-Environment and Resources, Chinese Academy of Sciences, Lanzhou 730000, China; wei_liu_xuan@yeah.net (X.W.); ygj7518@163.com (G.Y.); wy_2018dream@yeah.net (Y.W.); chenyong@lzb.ac.cn (Y.C.); 2 University of Chinese Academy of Sciences, Beijing 100049, China; 3 Institutes of Science and Development, Chinese Academy of Sciences, Beijing 100190, China

Due to an error during production, the order of affiliation in published paper [1] was incorrect.

**Incorrect:**

**Xuan Wei** **^1,2^, Lihua Zhou** ^2,3,^*, Guojing Yang ^1^, Ya Wang ^1^ and Yong Chen ^1^

^1^  Northwest Institute of Eco-Environment and Resources, Chinese Academy of Sciences, Lanzhou 730000, China; wei_liu_xuan@yeah.net (X.W.); ygj7518@163.com (G.Y.); wy_2018dream@yeah.net (Y.W.); chenyong@lzb.ac.cn (Y.C.)
^2^  Institutes of Science and Development, Chinese Academy of Sciences, Beijing 100190, China; lhzhou@casisd.cn
^3^  University of Chinese Academy of Sciences, Beijing 100049, China
*  Correspondence: lhzhou@casisd.cn

**Correct:**

**Xuan Wei** **^1,2^, Lihua Zhou** ^2,3,^*, Guojing Yang ^1^, Ya Wang ^1^ and Yong Chen ^1^

^1^  Northwest Institute of Eco-Environment and Resources, Chinese Academy of Sciences, Lanzhou 730000, China; wei_liu_xuan@yeah.net (X.W.); ygj7518@163.com (G.Y.); wy_2018dream@yeah.net (Y.W.); chenyong@lzb.ac.cn (Y.C.)
^2^  University of Chinese Academy of Sciences, Beijing 100049, China
^3^  Institutes of Science and Development, Chinese Academy of Sciences, Beijing 100190, China
*  Correspondence: lhzhou@casisd.cn
